# Bromelain Modulates Liver Injury, Hematological, Molecular, and Biochemical Perturbations Induced by Aluminum via Oxidative Stress Inhibition

**DOI:** 10.1155/2022/5342559

**Published:** 2022-11-21

**Authors:** Fatma M. El-Demerdash, Doha M. Hussien, Nora F. Ghanem, Ammar M. AL-Farga

**Affiliations:** ^1^Department of Environmental Studies, Institute of Graduate Studies and Research, University of Alexandria, Alexandria, Egypt; ^2^Department of Zoology, Faculty of Science, Kafr ElSheikh University, Kafr El-Sheikh, Egypt; ^3^Department of Biochemistry, Jeddah University, Saudi Arabia

## Abstract

Aluminum (Al) is an important factor in the environment as it is used in agriculture and several industries leading to hazardous effects via oxidative stress. Bromelain is a cheap extract from the byproduct waste of *Ananas comosus* stem. It has been used in several biological and therapeutic applications. So, this study was undertaken to assess the hepatoprotective potential of bromelain versus oxidative stress induced by aluminum chloride in rats. Results revealed that administration of AlCl_3_ reduced the body and liver weights and increased Al concentration in the blood and liver tissue. Also, AlCl_3_ caused valuable changes in hematological parameters and increased TBARS and H_2_O_2_ concentrations in rat liver. Enzymatic (SOD, CAT, GPx, GR, and GST) and nonenzymatic (GSH) antioxidants and protein content were significantly decreased. Furthermore, alterations in liver biomarkers such as bilirubin level and enzyme activities in both serum and liver homogenate (LDH, ALP, AST, and ALT) were detected. AlCl_3_ also caused inflammation as indicated by upregulation of the inflammation-related genes [interleukin 1 beta (IL-1*β*)], tumor necrosis factor-alpha (TNF-*α*), as well as matrix metalloproteinase (MMP9), and downregulation of nuclear factor erythroid 2 (Nrf2) expression. In addition, histopathological examination showed significant variations in the liver that confirms the biochemical results. Otherwise, bromelain intake alone slumped lipid peroxidation and gotten better antioxidant status significantly. Moreover, supplementation with bromelain before AlCl_3_ intoxication restores enzymatic and nonenzymatic antioxidants as well as biochemical indices and tissue architecture with respect to the AlCl_3_ group. In conclusion, bromelain proved its remarkable protective power to abolish AlCl_3_ toxicity. So, it might represent a new strategy in the therapy of metal toxicity by its antioxidant capacity.

## 1. Introduction

Aluminum (Al) is an important metal widely disseminated in various environmental compartments and largely used in daily life leading to many health problems in humans and animals [1]. The major origin of Al is due to its ingestion in different food (corn, yellow and processed cheese, baking powder, and flour) [2, 3]. Aluminum and its different compounds are used in the food industry (processing, packaging, and storage) leading to an increase in their levels in foods [4]. Also, they are used broadly in water purification [5], in medicines such as antacids, and in food additives authorizing their entrance into the body [6] and causing serious health problems in human and animal [7]. As an environmental contaminant, Al exposure generated hurtful effects on different biological systems including blood constituents, nervous, respiratory, skeletal, and immune systems [7]. Additionally, it is of great importance in various free radical-mediated diseases, osteomalacia, nephrotoxicity, and hepatotoxicity [8, 9]. Aluminum toxicity occurred through different mechanisms that encompass increasing blood-brain barrier permeability, interference with phosphorylation-dephosphorylation processes, and interchange of ions metabolism with successive free radicals' production and perturbation of the second messenger system [10]. Al salts may affect enzyme activity like hexokinase, phosphatases, phosphodiesterase, and phosphooxydase [5, 11]. Moreover, Al generates reactive oxygen species (ROS) [12], resulting in oxidative deterioration of lipids, proteins, and DNA.

Great interest is directed to many plants because of their antioxidant potential. *Ananas comosus* (Pineapple), which belongs to the Bromeliaceae family, is one of them. Waste usage is a promising strategy to get rid of the huge waste from processing. *Ananas comosus* is largely cultivated in the equatorial regions worldwide and has wide beneficial known effects as antioxidant, anticancer, anti-inflammatory, and antiplatelet impact. *A. comosus* stem extract is a waste product rich in complex enzymes identified by bromelain which are so important in some clinical applications, especially tumor growth modulation, wound healing, anti-inflammatory effect, antidiarrhea, and digestive help [13–15]. Bromelain has many commercial uses including food industry, pharmaceutical products such as cosmetics, for health benefits, and supplements as well as protein hydrolysates production [16, 17]. Prolonged oral use of bromelain is safe and it can be absorbed easily in the human intestinal tract without any decomposition or activity loss [18, 19]. The utilization of pineapple wastes as a source of bioactive compounds, especially proteolytic enzymes, is an alternative means. Therefore, the present study was designed to assess the potential antioxidant role of bromelain in modulating the harmful impacts induced by aluminum in male rats.

## 2. Materials and Methods

### 2.1. Chemicals

Bromelain from *A. comosus* (Pineapple) stem extract (600 GDU's/g) was purchased from Holland and Barrett, England. Aluminum chloride (AlCl_3_) was bought from Aldrich Chemical Company (Milwaukee, USA).

### 2.2. Experimental Design

The experimental design was performed following the US National Institute of Health Guidelines for the Care and Use of Laboratory Animals and Helsinki's declaration of animal ethics as approved by the Research Ethics Committee of Alexandria University (AU14-200204-1-3). Twenty-eight male Wister rats (150–170 g) were bought from the Faculty of Medicine, Alexandria University, Alexandria, Egypt. Rats were distributed randomly in cages seven per each and kept on a commercial diet and provided with tap water *ad libitum* and acclimated for two weeks (temperature, 21°C; photoperiod, 7 a.m. to 7 p.m.). Animals were classified into four groups: control, bromelain (250 mg/kg), AlCl_3_ (34 mg/Kg, 1/25 LD_50_), and bromelain plus AlCl_3_, respectively. Bromelain was administered one hour before AlCl_3_ intoxication daily while AlCl_3_ was given day after day orally for 30 days according to Saxena and Panjwani and El-Demerdash [20, 21], respectively. None of the AlCl_3_-intoxicated rats showed signs of morbidity or mortality during the study. At the experiment termination, rats were anesthetized using isoflurane and then killed via cervical dislocation, and livers were immediately removed. The liver was divided into two portions: the first portion was fixed in 10% formalin for histopathology examination, and the second portion was stored at -80°C for biochemical analyses.

### 2.3. Measurement of Aluminum Concentration

The level of AlCl_3_ was measured in the blood and liver tissue according to the method of Van Ginkel et al. [22] using the atomic absorption spectrometer (Shimadzu, AA6200).

### 2.4. Blood Samples

Complete blood counts (CBC) were performed in the collected blood samples by automatic methods (Sysmex kx-21n automated hematology analyzer; JAPAN CARE CO., LTD) including hemoglobin (Hb), white blood cells (WBCs), red blood cells (RBCs), platelets and hematocrit, or packed cell volume (PCV). Other blood samples were assembled for serum preparation and were left in a stand position for 30 min for blood clotting at 25°C then centrifuged at 3000 g for 15 min. The serum of each sample was taken and stored at -80°C till utilized in the determination of biochemical parameters.

### 2.5. Tissue Preparation

Livers were taken away and homogenized in ice-cold 0.01 mol/l sodium-potassium phosphate with 1.15% KCl buffer (pH 7.4). The homogenate was centrifuged at 10,000 g (4°C) for 20 min then the supernatants were taken and utilized for the determination of different assays.

### 2.6. Determination of TBARS, H_2_O_2_, and Glutathione Content

Thiobarbituric acid-reactive substances (TBARS), hydrogen peroxide (H_2_O_2_), and reduced glutathione (GSH) content were determined using the methods of Ohkawa et al., Velikova et al., and Ellman, respectively [23–25].

### 2.7. Determination of Antioxidant Enzyme Activities

The activities of superoxide dismutase (SOD; EC 1.15.1.1), catalase (CAT; EC 1.11.1.6), and glutathione S-transferase (GST; EC 2.5.1.18) were assessed by the methods of Misra and Fridovich, Aebi, and Habig et al. [26–28], respectively. While the activities of glutathione peroxidase (GPx; EC 1.11.1.9) and glutathione reductase (GR; EC 1.6.4.2) were evaluated according to Hafeman et al. [29].

### 2.8. Determination of Liver Function Biomarkers

Lactate dehydrogenase (LDH; EC 1.1.1.27) and alkaline phosphatase (ALP; EC 3.1.3.1) activities, protein content, and total bilirubin were estimated according to the methods used in the previous research [30–33]. Alanine aminotransferase (ALT; EC 2.6.1.2) and aspartate aminotransferase (AST; EC 2.6.1.1) activities were assayed using kits from Biodiagnostic, Egypt.

### 2.9. Molecular Analysis by Real-Time PCR

Using the RNeasy mini kit (Qiagen), total RNA was extracted from liver tissue in accordance with the manufacturer's recommendations. 10 *μ*g of RNA were reversely transcribed to produce first-strand cDNA. Real-time PCR was used to evaluate the relative expressions of matrix metallopeptidase 9 (MMP9), nuclear factor erythroid 2 (Nrf2), interleukin 1*β* (IL-1*β*), and tumor necrosis factor-*α* (TNF-*α*). Gene-specific primers are displayed in Table 1. A Real-Time PCR System (Applied Biosystems, USA) was used for the procedure, which consisted of 40 cycles of denaturation at 95°C for 30 s, annealing for both genes at 59°C for 30 s, and extension at 72°C for 30 s. The results were normalized to glyceraldehyde-3-phosphate dehydrogenase (GAPDH) mRNA expression that acts as the internal control and was amplified in the same process [34]. The relative gene expression was calculated using the 2^−∆∆Ct^ method.

### 2.10. Histopathological Examinations

Livers were fixed in 10% formalin and serial paraffin sections were obtained to examine the histological changes using hematoxylin and eosin stain [35] then, slides were photographed by light microscope (Olympus BX 41, Japan).

### 2.11. Statistical Analysis

Data from different groups were presented as means ± standard errors (SEM) and then analyzed utilizing SPSS software (version 22, IBM Co., Armonk, NY). Comparison between groups was performed by ANOVA followed by Tukey's post-hoc test. *P* value ≤0.05 was approved to be significant.

## 3. Results

### 3.1. Body Weight

**Figure 1:**
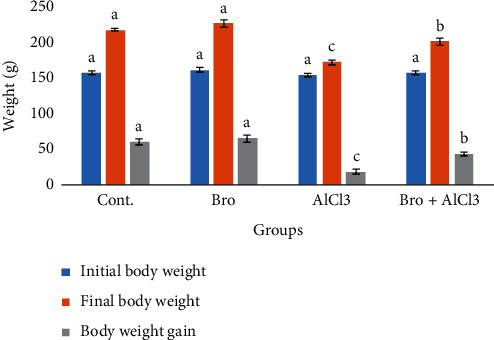
Initial and final body weights and body weight gain in rats of different experimental groups. Values are expressed as means ± SEM; *n* = 7 for each treatment group. A significant difference between the groups was shown with different superscript letters (a, b, and c), *P* < 0.05.

**Figure 2:**
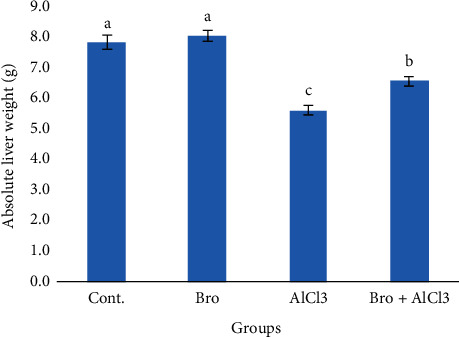
Absolute liver weight in rats of different experimental groups. Values are expressed as means ± standard error (SEM); *n* = 7 for each treatment group. Values are expressed as means ± SEM; *n* = 7 for each treatment group. A significant difference between the groups was shown with different superscript letters (a, b, and c), *P* < 0.05.

Final body weight and body weight gain in addition to the absolute liver weight of AlCl_3_-treated rats were significantly decreased as compared to control. However, bromelain supplementation alleviated this reduction with respect to AlCl_3_ exposed group. Bromelain alone did not cause any significant change (Figures 1 and 2).

### 3.2. Aluminum Concentration in Rat Liver and Blood

**Figure 3:**
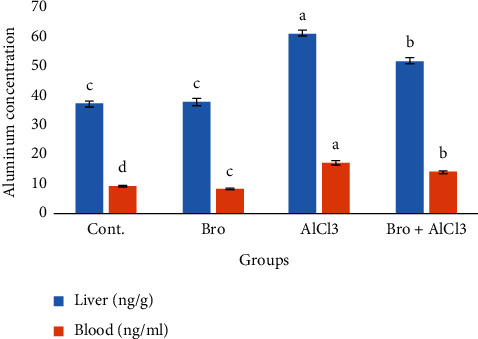
Aluminum concentration in liver and blood of male rats in different experimental groups. Values are expressed as means ± standard error (SEM); *n* = 7 for each treatment group. Values are expressed as means ± SE; *n* = 7 for each treatment group. A significant difference between the groups was shown with different superscript letters (a, b, c, and d), *P* < 0.05.

The Al concentration in rat liver and blood was measured after one month of oral AlCl_3_ administration (Figure 3). The level of Al in the liver and blood of the AlCl_3_ intoxicated group was increased by +64.21% and +81.45% when compared to the control group, respectively. However, this concentration was significantly decreased in the liver and blood of rats treated with bromelain plus AlCl_3_ by +38.56% and +47.89% as compared to the AlCl_3_ intoxicated group, respectively.

### 3.3. Hematological Parameters

Rats administered AlCl_3_ exhibited a significant decline in RBCs, Hb, PCV, and lymphocytes concentration while WBCs, platelets, and neutrophils increased significantly as compared to the control group. Other blood parameters are not significantly changed. On the other hand, the administration of bromelain alone showed a nonsignificant change in blood parameters as compared to the control group. Rats pretreated with bromelain and then received AlCl_3_ showed significant restoration near the normal level as compared to AlCl_3_ treated rats (Table 2).

### 3.4. Lipid Peroxidation and Reduced Glutathione Content

Results revealed that the levels of TBARS and H_2_O_2_ were significantly (*P* < 0.05) increased in rats treated with AlCl_3_ versus control while rats pretreated with bromelain and then intoxicated by AlCl_3_ presented a significant reduction in TBARS and H_2_O_2_ levels as compared to AlCl_3_ -treated rats. Otherwise, GSH content was significantly decreased in AlCl_3_ -treated rats. While in the rats' group ingested with both bromelain and AlCl_3_, induction in GSH content was observed as compared with AlCl_3_-treated rats. Supplementation with bromelain alone reduced the concentrations of TBARS and H_2_O_2_ and induced GSH content in liver homogenate significantly (Table 3).

### 3.5. Antioxidant Enzymes

A significant reduction (*P* < 0.05) in SOD, CAT, GPx, GR, and GST activity was observed in liver homogenate of AlCl_3_-treated rats. Furthermore, rats taken with bromelain+AlCl_3_ showed significant alleviation in antioxidant enzyme activities as compared to AlCl_3_-treated ones (*P* < 0.05). Moreover, the treatment of rats with bromelain alone improved antioxidant enzyme activities significantly versus the control group (Table 4).

### 3.6. Liver Function Biomarkers

Data showed that AST, ALT, and ALP activities were significantly (*P* < 0.05) decreased in liver homogenate and increased in rat serum while LDH activity increased in serum and liver homogenates of rats received AlCl_3_ with respect to control. Protein content was decreased while bilirubin was increased in rat liver homogenate, significantly. Moreover, a significant modulation in enzyme activities, protein and bilirubin contents in rats that received bromelain and then intoxicated with AlCl_3_ versus AlCl_3_ group was observed. Bromelain supplementation alone had insignificantly affected the measured parameters (Table 5).

### 3.7. Genes Expression

**Figure 4:**
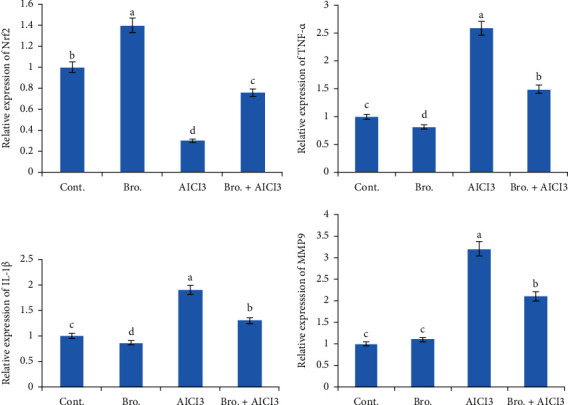
Effect of bromelain and aluminum on the gene expression (IL-1*β*, TNF-*α*, Nrf2, and MMP9) in rat liver. Data were normalized to the housekeeping gene (GAPDH). Data are presented as fold change (mean ± SEM; *n* = 7/group). Groups with different letters are significantly different at *P* < 0 : 05.

In the hepatic tissues of rats given AlCl_3_, a considerable upregulation in the mRNA expression of the MMP9, IL-1*β*, and TNF-*α* genes and a downregulation of the Nrf2 gene were found in comparison to the control group. On the other hand, the administration of bromelain prior to AlCl_3_ resulted in a considerable modification in the examined genes as opposed to the AlCl3 group. Furthermore, the intake of bromelain alone increased the Nrf2 mRNA expression in the hepatic tissues in comparison to the control group (Figure 4).

### 3.8. Liver Histopathology

**Figure 5:**
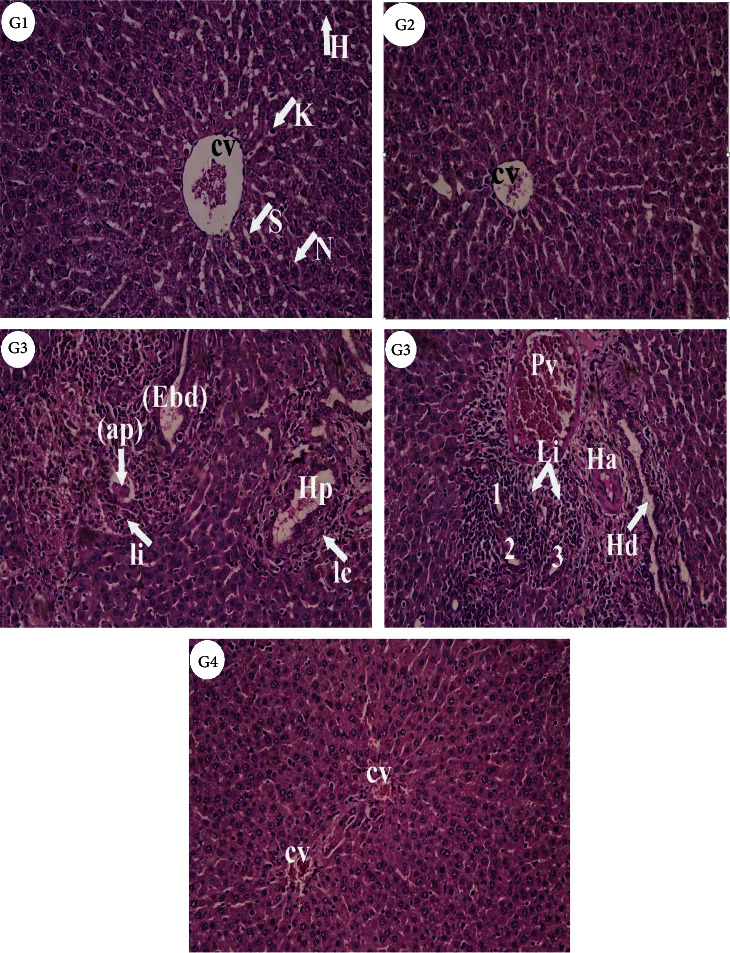
Photomicrographs of liver sections from different experimental groups stained with Hematoxylin & Eosin. Control **(G1)** and bromelain **(G2)** rats revealed the normal histological structures of normal hepatocytes (H) with nuclei (N), central vein (CV), sinusoids (S), and Kupffer cells (K) which represented monocytes-macrophage defense system. **(G3),** AlCl_3_ treated rats showed inflammation or infiltration (Ii) and enlarged bile ductular (Ebd), apoptic body (ap), branches of hepatic portal vein (Hp) surrounded by inflammatory cells (Ic). Also, leukocytic infiltration (Li) and bile ductular proliferation (1, 2, 3), branch of hepatic artery (Ha), congested portal vein (Pv) and enlarged hepatic duct (Hd) and hyperchromatic nuclei (Hn) were observed. **(G4)**, Bromelain+AlCl_3_ group revealed more or less normal hepatocytes (H&E X 200).

Light microscopic examination of the liver from control (G1) and bromelain (G2) groups showed normal architecture of hepatic lobules. However, liver sections of the AlCl_3_ group (G3) revealed enlarged and congested veins, bile ductular proliferation, leukocytic infiltration, activated Kupffer cells, and hepatocytes with pyknotic nuclei. While gradual improvement was observed in liver sections of rats treated with Bromelain+AlCl_3_ group (G4). Most hepatocytes appeared more or less normal, while some of them still showed cytoplasmic vacuolation (Figure 5).

## 4. Discussion

In the current investigation, the hepatoprotective role of bromelain from *Ananas comosus* stem against AlCl_3_ induced oxidative injury, and also hematological and biochemical perturbations were studied. To our knowledge, few references have indicated the adequacy of bromelain as a natural antioxidant to get rid of xenobiotic toxicity. AlCl_3_ administration impaired weight gain over the experimental period [36] due to malnutrition induced by the decrease in food consumption or by the toxicity induced by xenobiotics [37]. Also, a significant increase in Al concentration in blood and liver tissue was observed [38–40] in rats that received AlCl_3_ leading to calcium replacement [41]. Additionally, Al can bind to transferrin and some low molecular weight compound in the blood as citrate [42]. So, it interferes with Fe homeostasis by displacing it from transferrin leading to disturbance in iron metabolism [43]. Interestingly, bromelain supplementation for 30 days improved these parameters due to its antioxidant and chelation properties.

Blood and hematopoietic tissues rank as target organs for toxic effects of environmental chemicals; hence, they offer sensitive and reliable indicators, which could be effectively used to detect the magnitude of biochemical stress [44]. In agreement with the present results, perturbations in blood parameters in rats treated with AlCl_3_ were observed and this attributed to the inhibition of erythropoiesis and iron metabolism as well as alterations in erythrocyte morphology leading to anemia [45]. Furthermore, leukocytosis observed indicates the improved defense mechanism and immune system against infection induced by xenobiotics [46]. Bromelain is extensively used to improve tissue regeneration [47] and acts as an anti-inflammatory agent [19]. In this work, the administration of bromelain markedly hampered the toxic action produced by AlCl_3_ on hematological parameters as it is efficient in improving blood circulation and the amendment of arterial diseases [48]. Also, bromelain is used as a natural blood thinner because it prohibits exaggerated stickiness of blood platelets [49] and affects blood coagulation by inducing fibrinolytic capability and by reducing fibrin synthesis [50].

The liver is an important organ rich in mitochondria and plays a significant role in the metabolic process. AlCl_3_ inhibits the enzymes of oxidative phosphorylation leading to the cessation of energy metabolism [51]. So, oxidative stress and excess ROS production, via the Fenton reaction, have been involved in the mechanism of aluminum toxicity [21, 52] causing hepatocellular damage, apoptosis, and cellular necrosis [53, 54]. ROS Overproduction provokes injury via oxidizing cellular macromolecules such as lipids and proteins and triggering DNA injury [55]. Lipid peroxidation is a highly damaging peroxidative process that occurs in phospholipid compartments of the cellular membrane following metal intoxication [56]. AlCl_3_-treated rats manifested disruption in the antioxidant status where significant elevation in TBARS and H_2_O_2_ accompanied by a reduction in enzymatic (SOD, CAT, GPx, GR, and GST) and nonenzymatic antioxidants (GSH) in the liver homogenate were observed. The level of LPO is specified by the oxidants/antioxidants balance where the oxidants produced can be eliminated by the antioxidants [57, 58]. In agreement, previous authors showed that heavy metals work as oxidants affecting different organs leading to oxidative stress [8, 21, 54, 59–61]. On the other hand, treatment with bromelain given rise to a significant amelioration in oxidative stress markers (TBARS and H_2_O_2_) in AlCl_3_ intoxicated rats and this reflects its immense antioxidant properties and its interaction with heavy metals [8, 62, 63] beside the presence of cysteine, an amino acid with known antioxidant properties. Also, it is an important precursor in the output of glutathione, which protects cells from toxins as free radicals incriminated in AlCl_3_ toxicity [64]. Thus, bromelain supplementation could overcome AlCl_3_-induced hepatotoxicity by abolishing oxidative tissue injuries.

Glutathione is a low molecular weight tripeptide with a thiol group. It plays a significant function in cell metabolism and defense versus toxicants [57]. Glutathione can directly remove free radicals or provide detoxification using GSH-dependent enzymes (GST, GR, and GPx) as substrates [65]. Antioxidant enzymes (SOD, CAT, GPx, GR, and GST) have a majestic role in the elimination of ROS and keeping cellular homeostasis for normal cell function as well as act as indicators of oxidative stress [66]. The significant reduction in antioxidant enzymes and GSH might be attributed to Al accumulation observed in liver cells leading to a decline in enzyme protein synthesis [59, 67]. Superoxide dismutase is implicated in the cellular defense versus oxidative injury in aerobic living organisms, where it stimulates the conversion of superoxide anion to O_2_ and H_2_O_2_, which is decomposed by catalase into H_2_O. GPx protects the membrane lipids from oxidative injury [68] and catalyzes the reaction of hydroperoxide radicals with GSH to form disulfide glutathione (GSSG) [69]. While GST is a detoxifying enzyme that acts to convert xenobiotics into water-soluble nontoxic metabolites easily excreted outside the body [70]. Aluminum may affect the synthesis of GSH through the inhibition of glutathione-synthase and glucose 6-phosphate dehydrogenase activities. Moreover, it retards the conversion of oxidized glutathione (GSSG) into its reduced form (GSH) via GR inhibition [71]. Therefore, the antioxidant defense system is so important in the protection against oxidative stress-induced by Al and disturbing antioxidant enzymes [21, 72] in rat liver via the prohibition of free radicals chain reaction. Otherwise, the observed induction in antioxidant enzyme activities might be related to the decline in radical's generation and accumulation that are prohibited by bromelain [60]. Furthermore, it can protect against the toxic effects of ROS either by preventing their formation or interrupting their attack, via scavenging the reactive metabolites [73]. In accordance, ethanolic extract of *A. comosus* peel positively improved the antioxidant status by quenching and detoxifying the radicals stimulated by a carcinogenic substance and isoproterenol-caused oxidative injury in rats, respectively [20, 69]. Also, escalation in GSH content helps in the detoxification of ROS, preservation of cell integrity, and cellular components versus oxidation via the glutathione redox cycle due to its reducing features.

Toxic substances are transformed in the liver into less harmful products leading to hepatocytes damage. In the current study, rats treated with AlCl_3_ showed significant variations in serum and liver ALP, ALT, AST, and LDH activities as well as total bilirubin and protein. These parameters are important biomarkers for hepatocellular damage [74] and its alterations pointed out hepatocytes damage that altered the transport function and membrane permeability as well as leakage of enzymes from the cells to the bloodstream indicating hepatotoxicity [74–76]. Also, lipid peroxidation has a fundamental role in the disruption of hepatocellular membrane integrity, leading to the leakage of cytoplasmic enzymes and this confirmed the possible mechanism of oxidative stress in liver injury induced by Al [77]. Lactate dehydrogenase was significantly increased in AlCl_3_ intoxicated rats and this agreed with El-Demerdash [21]. This induction may be related to cellular impairment leading to disturbance in the metabolism of carbohydrates and protein as well as energy depletion [78]. Alkaline phosphatase is a critical membrane-bound enzyme in the biological processes used as a biomarker for heavy metals toxicity. It is responsible for the detoxification, metabolism, and biosynthesis of macromolecules which are required for many biological functions. Moreover, the decline in ALP activity in liver homogenate is consistent with the findings of Ochmanski and Barabasz and Szilagyi et al. [5, 11] who referred the change in ALP to the disturbance in bone formation induced by Al in addition to the binding of Al with DNA and RNA, respectively. So, alterations in these enzymes' activity could be expected due to cellular necrosis of the liver, kidney, and lung [79]. Protein is an essential cellular component susceptible to damage by free radicals and its depression might be linked to exaggerated leakage via nephrosis [80] or may be related to a disturbance in protein anabolic and catabolic processes. The elevation in total bilirubin may be due to diminished liver uptake, conjugation, or prolonged bilirubin output from hemolysis [21]. Moreover, results showed that bromelain treatment attenuated hepatotoxicity induced by AlCl_3_ since it could maintain hepatocytes' integrity and minimize the liver injury caused by AlCl_3_. Generally, it appears that the effectiveness of bromelain as a hepatoprotector because of its high content of active ingredients (ananasate, beta-sitosterol, campesterol, chlorogenic acid, rutin, naringenin, bromelain, vitamin A, B and C, glycosides, and flavonoids) that have potent antioxidant and anti-inflammatory activities [62, 81].

In agreement with the present results, several authors noted that Al administration significantly increased tissue TNF-*α* and the rise in cytokine expression suggests that the prooxidant/antioxidant balance has been upset. They also showed that during hepatocyte damage, activated Kupffer cells release growth factors and cytokines that have an encouraging effect on stellate cell activation and proliferation. Additionally, they release inflammatory mediators (TNF-*α*, IL-1*β*) that cause inflammatory leukocyte infiltration [59, 82, 83]. MMP-2 and MMP-9, two proteins associated with cell migration, were dramatically upregulated by AlCl_3_. It has been discovered that human tissue inflammation increases MMP expression. MMPs have a role in the regulation of inflammatory mediators, which attract immune cells to injured tissues [84, 85]. The observed downregulation in the Nrf2 gene's expression level in the hepatic tissues of AlCl_3_-intoxicated rats is consistent with findings made by Yu et al. [86] and Othman et al. [87], who discovered that Nrf2 deregulation is linked to Al toxicity. One of our most intriguing findings was that giving bromelain before AlCl_3_ increased the Nrf2 gene's level of expression in rat liver tissue. This would imply that one of bromelain's primary defense mechanisms against AlCl_3_-induced toxicity is the activation of Nrf2. Furthermore, the bromelain's impact on Nrf2 expression in the liver of the treated rats coincided with the antioxidant enzymes' functions in this investigation.

Histopathological examination of liver sections of AlCl_3_ intoxicated rats showed several lesions and abnormalities in the hepatocytes as enlarged and congested veins, proliferation and leukocytic infiltration, activated Kupffer cells, and hepatocytes with pyknotic nuclei. This could be attributed to the oxidative toxicity induced by AlCl_3,_ which may have apparently led to severe alterations in liver architecture. Similar observations exhibited severe degenerative alterations in liver hepatocytes with hepatic cord derangement, intrahepatic hemorrhage and inflammatory cells infiltration, karyopyknosis and necrosis [51, 61], brain and testis [12, 88], and heart tissues [89] of rats treated with AlCl_3_. Here, the observed degenerative alterations in liver tissues may be related to lipid peroxidation and free radicals' accumulation along with perturbation in antioxidant status induced by AlCl_3_ in rats. So, the histological examination confirmed the biochemical results and proved to be a good marker in liver failure. Based on our results, bromelain can improve most of the studied parameters in AlCl_3_ intoxicated rats and could restore hepatocyte integrity and decrease liver damage. In general, it appears that the effectiveness of bromelain as a hepatoprotector in AlCl_3_ toxicity may be referred to its high antioxidant constituents.

## 5. Conclusion

In conclusion, the current study pointed out that aluminum chloride has the potency to cause liver dysfunction via oxidative injury, alterations in the antioxidant defense system, liver function biomarkers, as well as molecular and histopathological changes. Furthermore, bromelain from *A. comosus* stem supplementation before aluminum treatment restores its toxic effects by quenching, chelating, and detoxifying the free radicals. So, our findings suggested that bromelain had a powerful antioxidant effect and could be used to develop functional healthy foods with hepatoprotective effects against complications induced by AlCl_3_.

## Figures and Tables

**Table 1:** Forward and reverse primer sequence used in RT-PCR.

Gene	Forward primer	Reverse primer
MMP9	5′-GATCCCCAGAGCGTTACTCG-3′	5′-GTTGTGGAAACTCACACGCC-3′
Nrf2	5′-TTGTAGATGACCATGAGTCGC-3′	5′-ACTTCCAGGGGCACTGTCTA-3′
TNF-*α*	5′-GCATGATCCGCGACGTGGAA-3′	5′-AGATCCATGCCGTTGGCCAG-3′
IL-1*β*	5′-CACCTCTCAAGCAGAGCACAG-3′	5′-GGGTTCCATGGTGAAGTCAAC-3′
Housekeeping GAPDH	GGTGAAGGTCGGAGTCAACG	TGAAGGGGTCATTGATGGCAAC

**Table 2:** Effect of bromelain (Bro), aluminum chloride (AlCl_3_), and their combination (Bro+AlCl_3_) on the hematological parameters in rats.

Parameters	Groups			
	Cont.	Bro	AlCl_3_	Bro+AlCl_3_
RBCs (x 10^6^/*μ*L)	8.03^a^ ± 0.274	8.41^a^ ± 0.246	5.78^b^ ± 0.264	8.33^a^ ± 0.345
WBCs (x 10^6^/*μ*L)	10.97^b^ ± 0.193	11.51^b^ ± 0.418	13.23^a^ ± 0.387	11.96^b^ ± 0.340
Hemoglobin (g/dl)	13.20^a^ ± 0.205	13.82^a^ ± 0.455	9.51^c^ ± 0.197	11.79^b^ ± 0.202
Platelets (10^3^/*μ*L)	427^c^ ± 12.08	427^c^ ± 14.86	527^a^ ± 13.59	470^b^ ± 13.63
PCV (%)	47.16^a^ ± 1.65	47.16^a^ ± 1.42	38.10^c^ ± 1.07	42.09^b^ ± 1.25
MCV (fl)	63.58^a^ ± 1.90	66.28^a^ ± 1.99	65.80^a^ ± 1.99	65.47^a^ ± 2.11
MCH (pg)	18.90^a^ ± 0.620	19.18^a^ ± 0.487	19.90^a^ ± 0.528	19.47^a^ ± 0.603
MCHC (%)	32.59^a^ ± 0.298	32.46^a^ ± 0.301	31.93^a^ ± 0.506	31.99^a^ ± 0.415
Neutrophils (%)	19.14^b^ ± 0.459	19.7^b^ ± 0.522	23.29^a^ ± 0.837	20.71^b^ ± 0.680
Lymphocytes (%)	58.13^a^ ± 0.527	56.26^ab^ ± 2.15	45.36^c^ ± 1.20	52.75^b^ ± 1.28
Eosinophils (%)	1.57^a^ ± 0.297	1.60^a^ ± 0.170	1.54^a^ ± 0.177	1.58^a^ ± 0.276
Monocytes (%)	4.19^a^ ± 0.142	4.07^a^ ± 0.170	4.36^a^ ± 0.180	4.17^a^ ± 0.166

RBC: red blood cell; WBC: white blood cell; hb: hemoglobin; PCV: packed cell volume; MCV: mean cell volume; MCH: mean corpuscular hemoglobin; MCHC: mean corpuscular hemoglobin concentration. Values are expressed as means ± SEM; *n* = 7 for each treatment group. ^abc^ Mean values within a row not sharing a common superscript letter were significantly different, *P* < 0.05. Statistically significant variations are compared as follows: Bromelain and AlCl_3_ groups are compared vs the control group while Bromelain+AlCl_3_ group is compared vs the AlCl_3_ group.

**Table 3:** Effect of bromelain (Bro), aluminum chloride (AlCl_3_) and their combination (Bro+AlCl_3_) on the level of thiobarbituric acid reactive substances (TBARS), hydrogen peroxide (H_2_O_2_), and reduced glutathione (GSH) content in rat liver.

Parameters	Groups			
	Cont.	Bro	AlCl_3_	Bro+AlCl_3_
TBARS (nmol/g tissue) % change	30.20^c^ ± 0.899	23.49^d^ ± 0.599 (-22.22%)	44.49^a^ ± 1.28 (+47.33%)	36.31^b^ ± 0.928 (+20.25%)
H_2_O_2_ (*μ*mol/g tissue) % change	63.65^c^ ± 1.62	50.35^d^ ± 1.36 (-20.90)	90.26^a^ ± 1.45 (+41.81%)	78.92^b^ ± 2.02 (+23.98%)
GSH (mmol/mg protein) % change	1.72^b^ ± 0.052	2.05^a^ ± 0.061 (+19.37%)	0.99^d^ ± 0.031 (-42.27%)	1.39^c^ ± 0.041 (-18.95%)

Values are expressed as means ± SEM; *n* = 7 for each treatment group. ^abcd^ Mean values within a row not sharing a common superscript letter were significantly different, *P* < 0.05. Statistically significant variations are compared as follows: Bromelain and AlCl_3_ groups are compared vs control group while Bromelain+AlCl_3_ group is compared vs AlCl_3_ group.

**Table 4:** Effect of bromelain (Bro), aluminum chloride (AlCl_3_), and their combination (Bro+AlCl_3_) on the activities of antioxidant enzymes in rat liver.

Parameters	Groups			
	Cont.	Bro	AlCl_3_	Bro+AlCl_3_
SOD (U/mg protein) % change	75.93^b^ ± 2.25	91.38^a^ ± 3.19 (+20.35%)	38.83^d^ ± 1.16 (-48.86%)	60.46^c^ ± 1.64 (-20.37%)
CAT (*μ*mol/hr/mg protein) % change	46.07^b^ ± 1.62	54.85^a^ ± 1.82 (+19.05%)	25.39^d^ ± 0.574 (-44.89%)	35.72^c^ ± 1.08 (-22.47%)
GPx (U/mg protein) % change	1.07^b^ ± 0.038	1.26^a^ ± 0.041 (+18.08%)	0.60^d^ ± 0.021 (-43.66%)	0.85^c^ ± 0.025 (-20.66%)
GR (U/mg protein) % change	1.23^b^ ± 0.042	1.46^a^ ± 0.036 (+18.77%)	0.70^d^ ± 0.023 (-42.53%)	0.99^c^ ± 0.033 (-19.46%)
GST (*μ*mol/hr/mg protein) % change	1.26^b^ ± 0.032	1.50^a^ ± 0.045 (+19.23%)	0.67^d^ ± 0.022 (-46.93%)	0.99^c^ ± 0.027 (-21.22%)

Values are expressed as means ± SEM; *n* = 7 for each treatment group. ^abcd^ Mean values within a row not sharing a common superscript letter were significantly different, *P* < 0.05. Statistically significant variations are compared as follows: Bromelain and AlCl_3_ groups are compared vs control group while Bromelain+AlCl_3_ group is compared vs AlCl_3_ group.

**Table 5:** Effect of bromelain (Bro), aluminum chloride (AlCl_3_), and their combination (Bro+AlCl_3_) on enzyme activities and protein content in serum and liver of male rats.

Parameters	Groups			
	Cont.	Bro	AlCl_3_	Bro+AlCl_3_
**Serum**				
AST (U/l) % change	53.12^c^ ± 1.12	51.20^c^ ± 1.51 (-3.62%)	76.88^a^ ± 2.29 (+44.74%)	65.97^b^ ± 1.75 (+24.19%)
ALT (U/l) % change	58.57^c^ ± 1.28	54.70^c^ ± 0.84 (-6.61%)	82.89^a^ ± 2.42 (+41.54%)	71.55^b^ ± 2.37 (+22.17%)
LDH (U/l) % change	585^c^ ± 14.32	563^c^ ± 14.21 (-3.71%)	823^a^ ± 18.84 (+40.82%)	713^b^ ± 20.03 (+21.95)
ALP (U/l) % change	59.01^c^ ± 1.90	58.20^c^ ± 2.09 (-1.38%)	83.80^a^ ± 2.32 (+42.01%)	69.47^b^ ± 2.05 (+17.72%)
Bilirubin (mg/dl) % change	0.730^c^ ± 0.017	0.732^c^ ± 0.016 (+0.25%)	1.02^a^ ± 0.027 (+39.13%)	0.871^b^ ± 0.028 (+19.29%)
**Liver**				
AST (U/mg protein) % change	125^a^ ± 3.75	131.32^a^ ± 3.15 (+4.79%)	80.43^c^ ± 1.78 (-35.78%)	102^b^ ± 2.88 (-18.54%)
ALT (U/mg protein) % change	162^a^ ± 5.03	154^a^ ± 3.29 (-5%)	99^c^ ± 3.40 (-38.73%)	131^b^ ± 4.21 (-19.12%)
LDH (U/mg protein) % change	1001^c^ ± 34.04	940^c^ ± 31.17 (-6.07%)	1366^a^ ± 37.79 (+36.51%)	1206^b^ ± 27.83 (+20.58%)
ALP (U/mg protein) % change	344^a^ ± 9.98	374^a^ ± 11.18 (+8.87%)	216^c^ ± 6.21 (-37.32%)	291^b^ ± 7.68 (-15.33%)
Protein (mg/g tissue) % change	194^a^ ± 2.44	197^a^ ± 4.29 (+1.48%)	128^c^ ± 4.45 (-34.15%)	163^b^ ± 4.02 (+16.10%)

Values are expressed as means ± SEM; *n* = 7 for each treatment group. ^abc^ Mean values within a row not sharing a common superscript letter were significantly different, *P* < 0.05. Statistically significant variations are compared as follows: Bromelain and AlCl_3_ groups are compared vs control group while Bromelain+AlCl_3_ group is compared vs AlCl_3_ group.

## Data Availability

All data are incorporated in the manuscript.
